# Initiation of recombination suppression and PAR formation during the early stages of neo-sex chromosome differentiation in the Okinawa spiny rat, *Tokudaia muenninki*

**DOI:** 10.1186/s12862-015-0514-y

**Published:** 2015-10-29

**Authors:** Chie Murata, Yoko Kuroki, Issei Imoto, Masaru Tsukahara, Naoto Ikejiri, Asato Kuroiwa

**Affiliations:** Department of Human Genetics, Institute of Health Biosciences, Tokushima University Graduate School, 3-18-15, Kuramoto-cho, Tokushima Japan; RIKEN, Center for Integrative Medical Sciences, 1-7-22 Suehiro-cho, Tsurumi, Yokohama, Kanagawa Japan; Present address: Division of Pediatric Disease Genomics, Department of Genome Medicine, National Research Institute for Child Health and Development, 2-10-1 Okura, Setagaya-ku, Tokyo, Japan; Student Laboratory, Faculty of Medicine, Tokushima University, 3-18-15, Kuramoto-cho, Tokushima Japan; Laboratory of Animal Cytogenetics, Faculty of Science, Hokkaido University, Kita 10 Nishi 8, Kita-ku, Sapporo, Hokkaido Japan

**Keywords:** Sex chromosome, neo-X, neo-Y, Evolution, Pseudoautosomal region, Recombination suppression, Biased gene conversion, BAC, Next-generation DNA sequencing

## Abstract

**Background:**

Sex chromosomes of extant eutherian species are too ancient to reveal the process that initiated sex-chromosome differentiation. By contrast, the neo-sex chromosomes generated by sex-autosome fusions of recent origin in *Tokudaia muenninki* are expected to be evolutionarily ‘young’, and therefore provide a good model in which to elucidate the early phases of eutherian sex chromosome evolution. Here we describe the genomic evolution of *T. muenninki* in neo-sex chromosome differentiation.

**Results:**

FISH mapping of a *T. muenninki* male, using 50 BAC clones as probes, revealed no chromosomal rearrangements between the neo-sex chromosomes. Substitution-direction analysis disclosed that sequence evolution toward GC-richness, which positively correlates with recombination activity, occurred in the peritelomeric regions, but not middle regions of the neo-sex chromosomes. In contrast, the sequence evolution toward AT-richness was observed in those pericentromeric regions. Furthermore, we showed genetic differentiation between the pericentromeric regions as well as an accelerated rate of evolution in the neo-Y region through the detection of male-specific substitutions by gene sequencing in multiple males and females, and each neo-sex–derived BAC sequencing.

**Conclusions:**

Our results suggest that recombination has been suppressed in the pericentromeric region of neo-sex chromosomes without chromosome rearrangement, whereas high levels of recombination activity is limited in the peritelomeric region of almost undifferentiated neo-sex chromosomes. We conclude that PAR might have been formed on the peritelomeric region of sex chromosomes as an independent event from spread of recombination suppression during the early stages of sex chromosome differentiation.

**Electronic supplementary material:**

The online version of this article (doi:10.1186/s12862-015-0514-y) contains supplementary material, which is available to authorized users.

## Background

The eutherian Y chromosome was originally homologous to the X chromosome [1]. Since the acquisition of the sex-determining loci, however, the Y has progressively degenerated due to suppression of recombination with the X. In the early stages of sex-chromosome differentiation, it is suggested that interactions between sex-determining genes and sexually antagonistic genes can drive selection for reduced recombination via gradual reduction of crossover frequencies, due to the spread of genetic modifiers of recombination rates and/or chromosome rearrangements such as inversions [2]. A lack of recombination results in accumulation of more deleterious substitutions relative to recombining regions, ultimately leading to deletion of non-functional DNA segments from the Y chromosome [3].

Sex chromosomes have evolved independently many times from different autosomes in different lineages [4]. In each lineage, different features are associated with sex-chromosome evolution. *Drosophila* lacks recombination during male meiosis, so they have completely differentiated and largely degenerated Y chromosomes [5]. The sex chromosomes of some fishes and plants have not evolved large non-recombining regions because their independently- and repeatedly-formed sex chromosomes have not had time for recombination to be suppressed or as a consequence of extensive ongoing recombination along the most region of sex chromosomes over long periods of time, as also illustrated by the sex chromosomes of palaeognathous birds [6–9]. In contrast, most eutherians have evolved largely degenerated Y chromosomes with the minute homologous region known as the pseudoautosomal region (PAR), which derived from an autosomal pair fused to the sex chromosomes in eutherian ancestor [1]. In eutherian, chiasma formation between X and Y chromosomes in the PAR are essential for the correct segregation of the sex chromosomes in male meiosis [10, 11]. Obligate crossing over in the minute PAR results in an extremely high recombination rate in the region relative to other genomic region, and the high frequency of meiotic recombination is expected to cause an increase in G + C content within PAR through GC-biased gene conversion (gBGC) [12, 13]. Previous studies suggest that the rapid progression of Y gene decay occurred shortly after the initiation of the sex chromosome differentiation in eutherian [4, 14, 15]. Therefore, the eutherian sex chromosomes might diverge largely because of not only their ancient origin, but also an unrecognized mechanism that accelerates Y degeneration. It is needed to understand the both processes of recombination suppression and PAR formation during early sex chromosome differentiation for revealing the eutherian-specific feature in Y degeneration. However, it remains unknown when and how PAR is formed in the sex chromosomes. Sex chromosomes of most of the highly diverged extant eutherian species are too ancient to address this issue. By contrast, the neo-sex chromosomes generated by sex-autosome fusions of recent origin are expected to be evolutionarily young. Therefore, such neo-sex chromosomes provide a good model in which to elucidate the early phases of eutherian-specific sex chromosome evolution.

The Okinawa spiny rat (*Tokudaia muenninki*) has neo-sex chromosomes formed by sex-autosome fusions and is therefore an excellent model for studies of the initiation process of sex chromosome differentiation [16]. Cross-species fluorescence *in situ* hybridization (Zoo-FISH) has revealed that a pair of autosomes fused with the sex chromosomes of *T. muenninki* (neo-X and neo-Y), which are homologous to segments of chromosomes 11 and 16 of mouse [16]. The neo-sex chromosomes of *T. muenninki* correspond to autosomes in the two other *Tokudaia* species, *Tokudaia tokunoshimensis* and *Tokudaia osimensis* [17], indicating that the sex-autosome fusions occurred independently in the *T. muenninki* lineage after it diverged from the last common *Tokudaia* ancestor. On the basis of the sequence data for mitochondrial cytochrome *b* (Cyt*b*) and nuclear recombination activating gene 1 (*RAG1*), divergence times between *T. muenninki* and the two other species are estimated to be around 1.5–1.7 and 0.6–0.8 million years ago (MYA) ([18]; on the basis of the substitution rate of this gene in murids, 0.932 (Cyt*b*) and 0.078 (*RAG1*) per 12 MY; [19]), suggesting that autosomes fused to sex chromosomes at 1.5–1.7 MYA or more recently. *T. muenninki* (2*n* = 44, XX/XY) is the only species in the genus *Tokudaia* that has maintained the Y chromosome, probably through fusion with an autosome [16, 18]. The short and long arms of their X chromosome (Xp and Xq) consisted of autosome (neo-X) and ancestral X, respectively, and the X chromosome had a large centromeric heterochromatin [16, 18]. The short arm of Y chromosome (Yp) consisted of autosome (neo-Y) in almost region and minute ancestral Y at the pericentromeric region, and the long arm of Y chromosome (Yq) was heterochromatic region including many *SRY* pseudogenes [16, 18]. The other two spiny rats lost the whole Y chromosome, except for a small region translocated to a single X chromosome in males and females [20, 21].

In the study described here, we applied molecular cytogenetic and genetic approaches to reveal the processes of PAR formation and recombination suppression during early-stage evolution of eutherian sex chromosomes, using *T. muenninki* neo-sex chromosomes as a model. First, we used fluorescence *in situ* hybridization (FISH) in a *T. muenninki* male to examine loci order in the neo-X and neo-Y chromosomes, as well as identify their chromosomal rearrangements. To estimate recombination rates along the neo-sex chromosome, the fine approach is to construct a genetic map by genotyping, with a high density of markers and a large number of unrelated individuals [22]. In single-sperm typing and genetic maps of three-generation families, male and sex-specific recombination rates also can be estimated, respectively. However, it is difficult to correct sufficient samples for estimating recombination rates by above analysis because *T. muenninki* is endangered and has been protected by the Japanese government as a natural treasure since 1972. Therefore, we detected variations in G + C content, which is positively correlated with recombination activity [12, 13, 23], by substitution-direction analysis using 116 gene sequences from the peritelomeric, middle, and pericentromeric regions of *T. muenninki* neo-sex chromosomes, to reveal recombination activity in different chromosomal locations. We also studied male-specific substitutions to identify the genetically diverged region between the neo-X and neo-Y, using partial sequences of 21 genes on three different regions of the neo-sex chromosomes. Furthermore, we determined the neo-X– and neo-Y–linked sequences of 18 genes to elucidate the processes underlying their genetic differentiation. In this study, we show that recombination have been suppressed in the pericentromeric region while recombination have been activated in the peritelomeric region of neo-sex chromosomes, indicating PAR might have evolved during the early stage of sex chromosome differentiation.

## Results

### Conserved synteny between neo-sex chromosomes

**Fig. 1:**
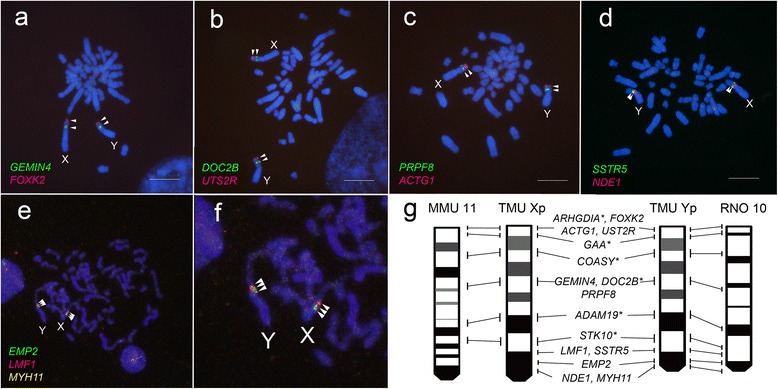
Two- and three-color FISH mapping of DAPI-stained chromosomes of a male *T. muenninki*. FITC-avidin (green)– and rhodamine-DIG (red)–labeled BAC DNA probes were used. Scale bar indicates 10 μm. Arrowheads indicate the locations of each BAC clone. Eleven genes, *GEMIN4* and *FOXK2* (**a**), *DOC2B* and *UTS2R* (**b**), *PRPF8* and *ACTG1* (**c**), *SSTR5* and *NDE1* (**d**), *EMP2*, *LMF1*, and *MYH11* (**e**, **f**) were mapped on *T. muenninki* neo-sex chromosomes. (**g**) Comparative cytogenetic maps from short arms of *T. muenninki* sex chromosomes (TMU Xp and Yp), mouse chromosome 11 (MMU 11), and rat chromosome 10 (RNO 10). The locations and order of the genes are shown on the side of each chromosome. The location of genes with asterisk and ideograms of *T. muenninki* neo-sex chromosomes were taken from Murata et al. (2012). The gene order and ideogram of MMU 11 and RNO 10 was taken from the NCBI database (http://www.ncbi.nlm.nih.gov/)

We performed two- and three-color FISH mapping, using bacterial artificial chromosome (BAC) clones of *T. muenninki* as probes, to confirm conserved synteny between their neo-sex chromosomes and the corresponding autosomes of mouse and rat. BAC clones used for FISH were screened based on genome information from mouse and rat, using as a reference the neo-sex–linked genes we identified in a previous study [16]. Sixteen, ten, and twenty four BAC clones were mapped to the peritelomeric, middle, and pericentromeric regions of both *T. muenninki* neo-X and neo-Y, respectively, as inferred from the genomes of the two other rodents (Fig. 1 and Additional file 1: Table S1). The neo-sex chromosomes were homologous to mouse chromosomes 11, 16 and 17, and to rat chromosome 10 (Additional file 1: Table S1). BAC FISH revealed no distinct chromosome rearrangements between the neo-sex chromosomes and rat chromosome 10 (Fig. 1).

### Substitution bias associated with G + C content in neo-sex chromosomes

We identified 51, 29, and 40 gene sequences located in the peritelomeric, middle, and pericentromeric regions of *T. muenninki* neo-sex chromosomes, respectively, from *de novo* assembled BAC sequences by performing the exon-intron definition based on the mapped read sequences to the mouse genome (Additional file 1: Table S2). Frameshifts were found in four genes on the neo-sex chromosomes (Additional file 1: Table S2), and the coding sequences of these genes were excluded from the following substitution-direction analysis.

To detect the variation in recombination frequency of *T. muenninki* neo-sex chromosomes, we estimated the G + C content in each of the three regions of the neo-sex chromosomes, which is positively correlated with recombination activity [12, 13]. We also estimated the frequency of nucleotide substitutions associated with the G + C content in the neo-sex chromosomes comparing to the corresponding autosomes in mouse and rat, which are the most closely related species to *T. muenninki* among the species with sufficient sequence data available for the analysis. Noncoding sites and third codon positions of coding sites, which are not generally subject to strong purifying selection, were used in this analysis. The mutation direction of each substitution was inferred in each species relative to the homolog nucleotide site in the other two species by the maximum parsimony principle. In the result, the G + C content was higher in the peritelomeric region and slightly lower in the pericentromeric region of *T. muenninki* neo-sex chromosomes than in the corresponding autosomal regions from mouse and rat (Table 1). Nucleotide substitutions at both the coding and noncoding sites were strongly biased toward A/T → G/C substitutions in the concatenated gene sequences from the peritelomeric region, but toward G/C → A/T substitutions in the pericentromeric region of *T. muenninki* neo-sex chromosomes (Table 1). This substitution bias was also observed in the separated gene sequences from each region (Additional file 2: Figure S1). By contrast, this strong bias in substitution direction was not detected in the middle region of *T. muenninki* neo-sex chromosomes or in any region of rat and mouse autosomes (Table 1). These results showed that the sequence evolution toward GC-richness and AT-richness had occurred in the peritelomeric and pericentromeric regions, respectively, through the strong substitution bias. In this analysis, the frequency of substitution direction in rat may be effected by some nucleotide substitutions occurred in the common ancestor of mouse and *T. muenninki*. Therefore, we also performed the substitution-direction analysis among *T. muenninki*, mouse, and rat with guinea pig as outgroup to compare *T. muenninki* with rat more properly (Additional file 1: Table S3). This result also showed the sequence evolution toward GC- and AT-richness in the peritelomeric and pericentromeric region of *T. muenninki* neo-sex chromosomes, respectively.

**Table 1:** Comparison of G + C content and nucleotide substitution frequency in three regions among *T. muenninki*, mouse, and rat

Species	Site	Genome category	No. of genes	No. of bases	GC content	No. of substitutions	A/T → G/C (%)	G/C → A/T	A/T → C/G	C/G → A/T	A/T → T/A	G/C → C/G
*T.muenninki*	neo-sex peritelomeric	ORF	50	22,288	69.5	1,452	51.9	25.1	9.8	2.8	3.6	6.9
*M.musculus*	chr. 11E1-E2				66.8	1,231	34.0	42.9	7.6	5.5	2.8	7.1
*R.norvegicus*	chr. 10q32.3				66.0	1,439	31.6	46.8	4.5	6.5	3.1	7.4
*T.muenninki*	neo-sex middle	ORF	29	15,596	64.7	655	35.9	42.3	6.7	5.2	4.4	5.5
*M.musculus*	chr. 11B5				65.3	791	36.9	37.3	11.0	4.7	4.2	5.9
*R.norvegicus*	chr. 10q24				64.2	957	32.0	45.4	7.1	6.3	3.3	6.0
*T.muenninki*	neo-sex pericentromeric	ORF	37	16,788	58.5	690	27.4	52.2	5.2	6.7	3.5	5.1
*M.musculus*	chr. 16A1-B3, chr. 17A3.3				59.6	839	37.1	40.6	7.5	5.1	3.9	5.7
*R.norvegicus*	chr. 10q11-12				59.4	1,150	36.3	41.1	7.0	5.7	4.9	5.0
*T.muenninki*	neo-sex peritelomeric	UTR + intron	51	342,340	52.4	22,385	40.3	24.9	13.4	6.3	7.3	7.8
*M.musculus*	chr. 11E1-E2				50.9	18,488	32.0	35.1	10.9	7.6	6.9	7.6
*R.norvegicus*	chr. 10q32.3				50.6	23,100	31.8	36.5	8.9	8.1	6.8	7.8
*T.muenninki*	neo-sex middle	UTR + intron	29	346,940	48.2	16,768	32.0	36.0	10.3	7.1	7.9	6.7
*M.musculus*	chr. 11B5				48.4	17,444	32.7	34.4	11.5	6.9	7.6	6.8
*R.norvegicus*	chr. 10q24				48.3	23,707	32.6	35.3	9.9	7.2	7.7	7.3
*T.muenninki*	neo-sex pericentromeric	UTR + intron	40	576,147	45.5	26,909	28.7	40.9	8.6	8.1	7.8	6.1
*M.musculus*	chr. 16A1-B3, chr. 17A3.3				46.2	29,346	32.2	34.8	11.3	7.2	8.0	6.4
*R.norvegicus*	chr. 10q11-12				46.4	46,550	34.4	33.1	10.5	7.2	7.9	6.9

We then inferred the substitution directions at all noncoding sites preceded by C or followed by G in three species to exclude the influence of DNA methylation on G + C content in the peritelomeric and pericentromeric regions of *T. muenninki* neo-sex chromosomes. The total rate of CpG → TpG and CpG → CpA substitutions due to deamination of 5-methyl-cytosine in methylated CpG pairs is estimated to be more than 10 times higher than those of other transitions [24, 25]. The total frequency of CpG → TpG and CpG → CpA substitution was only 3–3.5 % lower in the peritelomeric region and 0.3–0.7 % higher in the pericentromeric region in *T. muenninki* than in the corresponding autosomal regions of mouse and rat (Additional file 1: Table S4).

### Genetic differentiation between the pericentromeric regions of neo-sex chromosomes

We detected the male-specific substitutions to reveal the genetically diverged regions between neo-sex chromosomes by PCR and direct sequencing using genomic DNA from two-to-six males and four females. We found 47 male-specific substitutions and four indels in eleven of twelve partial gene sequences from the pericentromeric region, but no male-specific substitutions in all 12 partial gene sequences from the peritelomeric and middle regions (Additional file 1: Table S5 and S6). Each neo-sex chromosome-derived BAC clone was screened based on the inferred neo-X and neo-Y alleles described in Additional file 1: Table S6 to confirm the degree of genetic divergence between the neo-X and neo-Y. The open reading frames (ORFs) of 18 genes studied were conserved on both neo-sex chromosomes except for *TXNDC11*, although the complete ORF sequences were not determined in three genes. The neo-Y–linked sequence of *TXNDC11* contains the frameshift generated by a single base insertion, whereas its neo-X homolog does not (Additional file 2: Figure S2). Although neo-X derived BAC clone sequences did not be determined in *PRM1* and *CPPED1*, detection of male-specific substitutions revealed that the frameshift mutations occurred in these genes as well as *TXNDC11* in one allele of male (Additional file 1: Table S6).

**Fig. 2:**
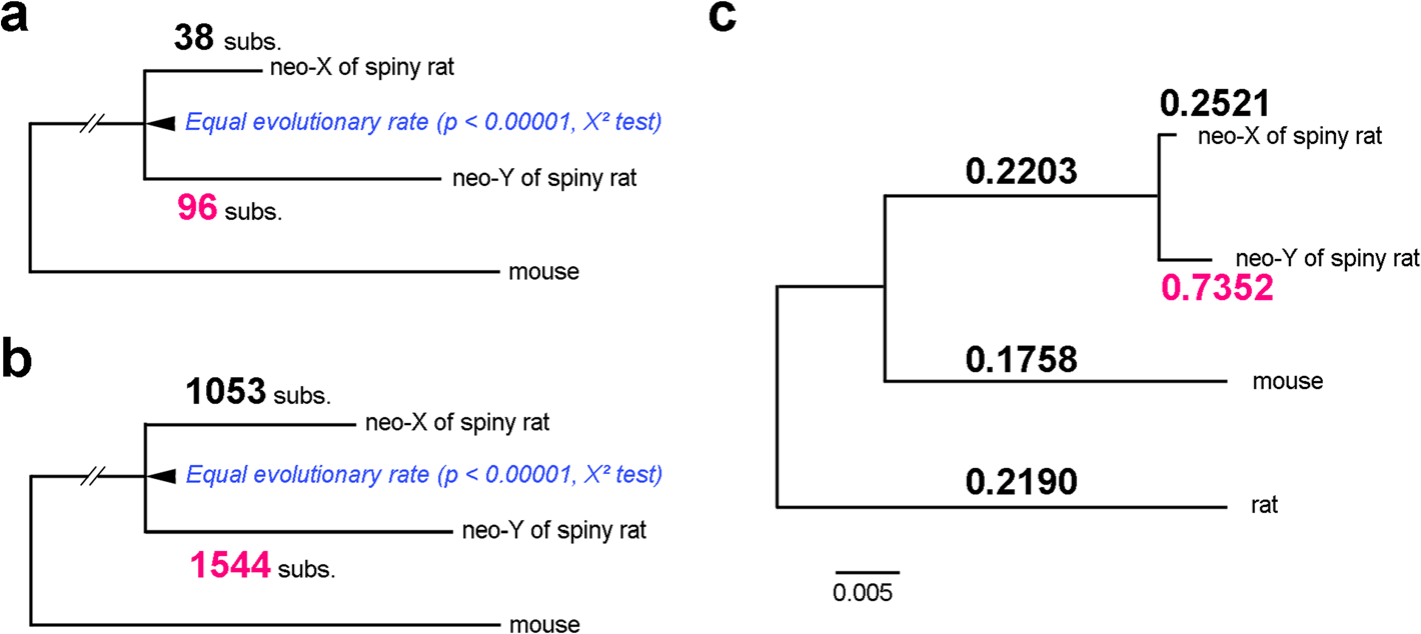
Sequence evolution of *T. muenninki* neo-sex chromosomes. **a** Nucleotide sequence evolution in the coding sites of 17 combined genes. **b** Nucleotide sequence evolution in the noncoding sites of 18 combined genes. Equality of the evolutionary rate between the neo-X and neo-Y was rejected based on a chi-squared test, and an accelerated rate of evolution in the neo-Y was demonstrated in both coding and noncoding sites. subs: substitutions. **c** Maximum likelihood tree of the coding sequences of 17 combined genes. Numbers above and under branches refer to d_N_/d_S_ ratios and maximum-likelihood bootstrap percentage (1000 replications), respectively. The free-ratio model is used, which assumes a different dN/dS ratio for each branch in the tree. d_S_: synonymous substitution rate; d_N_: non-synonymous substitution rate; spiny rat: *T. muenninki*; mouse: *M. musculus*; rat: *R. norvegicus*

The evolutionary rate was significantly higher in the neo-Y than the neo-X in the combined nucleotide sequences of all genes excluding *TXNDC11*, as well as in the noncoding sequences of all genes, comparing to mouse orthologues (Fig. 2a, b). The ratios of non-synonymous/synonymous substitution rates (d_N_/d_S_) for the combined 17 genes were estimated to reveal the variation in selection pressure on neo-Y genes using “free-ratio” model, which assumes a different d_N_/d_S_ ratio for each branch. We observed that d_N_/d_S_ ratio was 2.9 times higher in the neo-Y branch than neo-X branch, and the significant difference between neo-X and neo-Y branches was shown by likelihood ratio test (*P* = 0.0151, Fig. 2c and Additional file 1: Table S7). Furthermore, the neo-Y branch has significantly higher d_N_/d_S_ ratio than other branches, while no significant difference was found between the neo-X and other branches (*P* < 0.0001 and *P* = 0.5895, respectively, Additional file 1: Table S7).

As an additional analysis of substitution spectrum in the genetically diverged regions between neo-sex chromosomes, we estimated the frequencies of substitution directions in each neo-sex–derived noncoding sequence to distinguish between the neo-X and neo-Y by comparing with mouse sequence. Sequence evolution toward AT-richness was observed in both neo-X and neo-Y, although it was more pronounced on the neo-Y than the neo-X (Additional file 1: Table S8).

## Discussion

In agreement with a previous study [16], our FISH analysis detected no visible inversion and deletion between the neo-X and neo-Y in *T. muenninki* (Fig. 1). However, it remains the possibility of small rearrangements between them. The determination of the complete genomic sequence from each neo-X and neo-Y in the recombination suppressed region is needed to exclude the possibility. FISH analysis also showed the existence of homologous segment of mouse chr. 17 as well as chr. 11 and 16 in *T. muenninki* neo-sex chromosomes. The fluorescence signal of mouse chr. 17 probe on the neo-sex chromosomes would be too small to detect in Zoo-FISH analysis from our previous study [16]. It is hypothesized that a gradual reduction of crossover frequencies is due to the spread of genetic modifiers of recombination rates or to chromosome rearrangements such as inversions [2]. Recombination-suppressed sex chromosomes with chromosome inversions were reported in eutherian lineages. In humans, an autosome fused to the X chromosome (XAR) in the eutherian ancestor has high homology with the ancestral chromosome from which it is derived, whereas most of the autosome fused to the Y chromosome has genetically diverged from the XAR and then degenerated via at least eight inversions, leaving only the minute distal parts that recombine with the XAR during meiosis [1]. In black muntjac (*Muntiacus crinifrons*), recombination suppression due to a large autosomal inversion during the past 0.5 million years has led to the formation of the neo-Y diverged from the neo-X [26]. Compared to their sex-chromosomal regions, *T. muenninki* neo-sex chromosomes without distinct chromosome rearrangement are in a very early stage of sex-chromosome differentiation.

Over the course of sequence evolution, GC-richness increased in the peritelomeric region and decreased in the pericentromeric region of the neo-sex chromosomes (Table 1). G + C content is negatively correlated with the distance to telomeres [23]. However, distance from the telomere does not differ significantly between the three regions of *T. muenninki* neo-sex chromosomes and the corresponding regions of rat chromosome 10 (Fig. 1), suggesting that chromosomal location is not the cause of the specific substitution patterns in *T. muenninki* neo-sex chromosomes. Therefore, the sequence evolution toward GC- and AT-richness in the peritelomeric and pericentromeric region of neo-sex chromosomes, respectively, must be a consequence of genomic evolution from autosomes to sex chromosomes specifically in *T. muenninki* lineage. In human and bovine, G + C content of various regions can be ranked as follows (in decreasing order): PAR, autosomes, X chromosome, and Y chromosome; this ranking corresponds to decreasing order of recombination activity [12]. This effect of recombination is most probably a consequence of the neutral process of gBGC, i.e., a nonreciprocal exchange of nucleotide sequence that is biased in favor of GC-alleles resulting from mismatch repair of heteroduplex DNA formed during meiotic recombination [13, 23]. gBGC is expected to occur predominantly in recombination hotspots with short lifespans and weak influences on substitution rate, suggesting that it could affect the evolution of base composition in Mb-long genomic fragments, but not individual hotspots (typically 2 kb long) [13, 27]. This agrees well with our observation that G + C content was altered in both coding and noncoding sites of most of the genes we analyzed in the peritelomeric and pericentromeric regions (Additional file 2: Figure S1). We also observed sequence evolution toward AT-richness in both neo-X and neo-Y sequences from the pericentromeric regions that genetically diverged each other, and the bias toward AT-richness was stronger on the neo-Y than neo-X (Table 1 and Additional file 1: Table S8). In the pericentromeric region of neo-sex chromosome, recombination could be suppressed between neo-X and neo-Y and limited between neo-X chromosomes during female meiosis. Therefore, this region shows low recombination rate comparing to the corresponding autosomal region of other species and the other neo-X region recombining with neo-Y, in which recombination occurs during both male and female meiosis. This evolutionary pattern can be explained by a low gBGC effect due to depression of recombination activity that involved in change from autosomal inheritance to sex-linked inheritance. Variation in G + C content is also generated by other factors, such as large differences in the original G + C content or the DNA methylation frequency of CpG dinucleotides [24, 25, 28]. However, the observed G + C variation in *T. muenninki* neo-sex chromosomes cannot be explained by these two factors. Changes toward GC-richness and AT-richness were observed respectively in the peritelomeric region, which has high G + C content, and the pericentromeric region, which has low G + C content, relative to the corresponding autosomal regions in the two other species. There was little difference in the CpG → TpG/CpA substitution rate between *T. muenninki* and the other rodent species. Therefore, it was suggested that the sequence evolution toward GC-richness resulted from the increased gBGC by acquisition of recombination activity in the peritelomeric region of the neo-sex chromosomes. To reveal the recombination activity along the neo-sex chromosome, we need to estimate recombination rates in different regions by substitution-direction analysis as well as other approaches such as construction of a genetic map by genotyping with a high density of markers and a large number of unrelated individuals, single-sperm typing, and genetic maps of three-generation families, if we could correct sufficient number of DNA samples or fresh testis.

The lack of recombination results in accumulation of more substitutions on the Y chromosome than the X chromosome due to Muller’s ratchet, genetic hitchhiking, male-driven effects, positive selection, and/or relaxed purifying selection [3, 15, 29, 30]. In the pericentromeric region that genetically diverged between neo-sex chromosomes, we observed higher substitution rates in the neo-Y than neo-X (Fig. 2a, b, Additional file 1: Table S5 and S6), indicating that recombination had been suppressed in the pericentromeric region. We also observed higher d_N_/d_S_ ratio in neo-Y than neo-X lineage, suggesting that the neo-Y–linked genes had experienced relaxed purifying selection and/or positive selection (Fig. 2c and Additional file 1: Table S7). Consistent with this result, we detected the internal stop codon generated by the frameshift in the neo-Y–linked sequence of *TXNDC11*, but not the neo-X–linked sequence (Additional file 2: Figure S2). We also revealed that the frameshift mutations in *PRM1* and *CPPED1* as well as *TXNDC11* on the pericentromeric region were existed in one allele of all males (Additional file 1: Table S6), suggesting that the frameshift mutations were caused by neo-Y degeneration in the three genes. Further investigation is needed to identify factor accelerating the evolutionary rate on the neo-Y.

Our results suggested that the recombination was suppressed in the pericentromeric region of neo-sex chromosomes from any cause at least other than distinct chromosomal inversion (Figs. 1 and 2, Additional file 1: Table S6). This region locates most closely to the ancestral sex chromosomes among the three neo-sex regions [16], suggesting that recombination between the neo-X and neo-Y was suppressed due to gradually spread of genetic modifiers of recombination rates from the ancestral Y region to its neighboring region in the neo-Y chromosomes. Alternatively, it may be that the involvement of the heterochromatic arms is a prerequisite for recombination suppression, or can at least accelerate the process. In common mole-rat, *Cryptomys hottentotus natalensis* and *C. h. hottentotus*, asynapsis of X2 (autosomal homolog of Y) and Y (autosome fused to ancestral Y) occurs in 25 % of cells examined and that this involves their heterochromatic arms, while the sister taxon (*C. h. pretoriae*), which lacks these heterochromatic arms, displays complete synapsis of X2 and Y in 100 % of cells examined [31]. The asynapsis between the X2 and the Y leads to the absence of chiasma formation in this region, further emphasising that recombination is reduced between these chromosomes [31]. *T. muenninki* has an X chromosome containing a large pericentromeric heterochromatic region and a Y chromosome with heterochromatin in the proximal region of the short arm as well as the entire long arm [18]. Therefore, the involvement of pericentromeric heterochromatins in *T. muenninki* Xp and Yp may be prerequisite for recombination suppression, or can at least accelerate the process in their neo-sex chromosomes.

There is a significant difference between peritelomeric and middle regions in regard to the extent of sequence evolution toward GC-richness, of which both are recombining regions (Table 1, Additional file 1: Table S5). The result suggested that recombination activity had already been limited in the peritelomeric region of almost homologous neo-sex chromosomes, although in this study we could not study the recombination in male meiosis because they are endangered and protected species. The PAR has conserved synteny among most eutherian species in which it has been identified [32], suggesting that the PAR is formed during a very early stage of sex-chromosome differentiation. Therefore, acquirement of recombination activity limited in the peritelomeric region of sex chromosomes may be one of the mechanism that accelerates Y degeneration during a very early stage of sex chromosome differentiation in eutherian.

## Conclusions

**Fig. 3:**
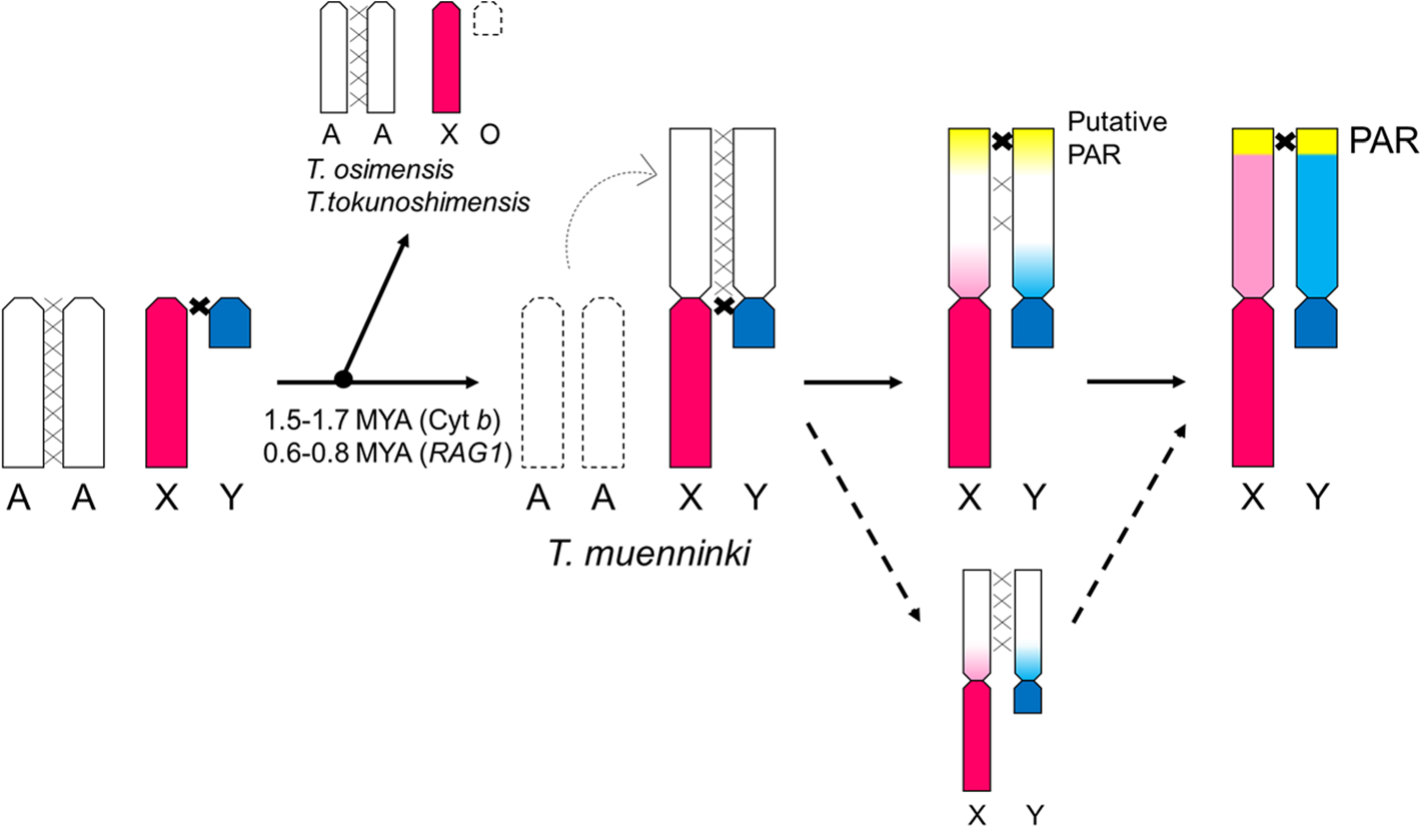
Evolution of neo-sex chromosomes. In the early stages of sex-chromosome differentiation, a putative PAR with recombination activity might have been formed prior to the spread of recombination suppression. Red: ancestral X region; blue: ancestral Y region; pink: neo-X region diverged from neo-Y; sky blue: neo-Y region diverged from neo-X; white: undifferentiated neo-sex chromosomal region; yellow: putative PAR. MYA: million years ago

Our research showed that recombination might have been suppressed and activated in the pericentromeric and peritelomeric region of almost homologous neo-sex chromosomes without distinct chromosome rearrangement in *T. muenninki*. We conclude that the PAR might have been formed independently of the spread of recombination suppression at the same time as the initiation of recombination suppression during an early stage of sex-chromosome differentiation (Fig. 3). *T. muenninki* neo-sex chromosomes are excellent models for the very early stages of sex-chromosome differentiation, and further analysis of them should provide new insights into eutherian sex-chromosome evolution.

## Methods

### Sample collection, cell culture, and DNA extraction

*T. muenninki* is endangered (The IUCN Red List of Threatened Species; http://www.iucnredlist.org/) and has been protected by the Japanese government as a natural treasure since 1972 [33]. We performed sample collection with permission from the Agency for Cultural Affairs and the Ministry of the Environment in Japan. After a small piece was cut from the tip of the tail of spiny rats for our study, all the spiny rats were released at their capture sites. We carried out cell culture and DNA extraction as previously reported [18] and used genomic DNA from six males and four females of *T. muenninki* in this study. All the animal experiments in this study were approved by Institutional Animal Care and Use Committee of National University Corporation Hokkaido University and performed in accordance with the Guidelines for the Care and Use of Laboratory Animals, Hokkaido University. This article does not contain any studies with human participants performed by any of the authors.

### Construction of BAC libraries and screening of BAC clones

BAC libraries were constructed according to previously described procedures [34]. Cultured fibroblast cells were embedded in 1 % agarose gels, treated with *SacI*, and subjected to pulsed-field gel electrophoresis. DNA fragments ranging from 125 to 225 kb were isolated and ligated into vector pKS145. Transformation was carried out by electroporation using *Escherichia coli* DH10B as a host strain. Ampicillin-resistant transformants were collected and stored in 384-well plates. PCR primer sets were designed against *M. musculus* gene sequences located on chromosomes 11, 16, and 17 (GRCm38/mm10 provided from UCSC) and the orthologous sequences located on chromosome 10 of *R. norvegicus* (RGSC 5.0/rn5 and Rnor_6.0 provided from UCSC and NCBI, respectively). These primers were used to screen the *T. muenninki* BAC library using a two-step 3D PCR screening system [34]. The primer sequences are provided in Additional file 1: Table S1. The identities of positive clones were confirmed by PCR using a single colony to provide template DNA. Clones screened for FISH analysis and each neo-sex sequence determination are presented in Additional file 1: Table S1 and S9, respectively. High-grade BAC DNA was obtained using the Plasmid Mini kit (QIAGEN) following the instructions of the manufacturer with minor modifications.

### Chromosome preparation and FISH

Chromosome preparation and FISH were performed as previously described protocol with slight modifications [35]. All BAC clones of *T. muenninki* were hybridized to male chromosomes. After hybridization overnight at 37 °C, hybridized probes labeled with biotin 16-dUTP or Digoxigenin (DIG) 11-dUTP by nick translation (Roche) were detected with FITC-avidin or anti-DIG-rhodamine (Roche), respectively, and then stained with 4′, 6-diamidino-2-phenylindole (DAPI). The FISH signals were observed under an Olympus fluorescence microscope BX51 and BX53 (Olympus), and the images were captured with an EXi Aqua digital camera (QImaging) and DP73 (Olympus), respectively.

### Determination of BAC sequence by next-generation sequencing (NGS)

BAC sequence was determined by NGS on an Illumina HiSeq 1000 using a 100 bp paired-end protocol or an Illumina MiSeq using a 300 bp paired-end protocol. The DNA library was prepared from seven BAC pools (details shown in Additional file 1: Table S1, S8, S10, and S11) using TruSeq DNA Sample Prep v2 (Illumina), following the manufacturer’s instructions, with a unique identifier index added to each pool for multiplex sequencing.

### Data assembly

*De novo* assembly was performed after removing sequences not from *T. muenninki* and quality filtering of reads. First, all short reads of each index group were mapped to the DNA sequences of cloning vector pKS145 (AB013921) and *E. coli* (NC_010473) using the BWA 0.5.9 aligner with the default setting, and then the unmapped BAM file generated by SAMtools 0.1.16 was converted into a FASTQ file using bam2fastq 1.1.0 [36, 37]. The unmapped reads were then mapped to the mouse genome sequences (mm10) using the BWA 0.5.9 aligner given 20 % uniform base error rate to identify genes included in BAC sequences determined by NGS. Next, using FASTX-Toolkit 0.0.13 and PRINSEQ lite 0.20.3, reads with 80 % of bases at quality threshold Q20 (1 in 100 chance of incorrect base call) and those that were less than 81 bp after trimming distal sequences of less than Q20 were removed. Finally, *de novo* assembly was carried out across multiple k-mer values using Velvet 1.0.17 (Additional file 1: Table S10 and S11) [38].

### Sequence determination of neo-sex–linked genes

The assembled genomic sequences of *M. musculus* (GRCm38/mm10, released on December 2011) and *R. norvegicus* (RGSC 5.0/rn5, released on March 2012) were downloaded from UCSC Genome Bioinformatics (http://genome.ucsc.edu/) and NCBI (http://www.ncbi.nlm.nih.gov/). *De novo* assembled sequences of *T. muenninki* BAC clones were annotated by identifying each exon of gene sequences determined by the reference mapping to mouse genome. The accession numbers for 120 neo-sex–linked genes of *T. muenninki* are listed in Additional file 1: Table S2. The coding and noncoding sequences of each gene from *T. muenninki* were respectively aligned with the coding and noncoding sequences of homologous genes from both mouse and rat using ClustalX 2.1 (Additional file 3) [39]. Gaps were filled with the sequences determined by the reference mapping or Sanger sequencing.

### Estimation of G + C content and frequency of nucleotide changes

Custom written C scripts were used to estimate the G + C content and the frequency of each substitution direction in the aligned noncoding and coding sequences among *T. muenninki*, *M. musculus*, and *R. norvegicus*, with or without guinea pig (*Cavia porcellus*) as outgroup. The direction of nucleotide substitutions among the three sequences was inferred by the maximum parsimony principle shown in Additional file 2: Figure S3. At a given position, if *T. muenninki* had a G-allele and the other species shared an A-allele, the substitution direction was inferred to be from A to G in *T. muenninki*. An unaligned region and repetitive sequences were excluded from this analysis.

### Detection of male-specific substitution and testing the molecular evolutionary rate

Substitution detection was performed in partial sequences of seven genes from the peritelomeric region, five genes from the middle region, and twelve genes from the pericentromeric region of the neo-sex chromosome (Additional file 1: Table S5). PCR was carried out in a reaction volume of 10 μl containing 25 ng of template genomic DNA, 0.3 μM of each primer (Additional file 1: Table S5), 0.2 mM of each of the four dNTPs, and 0.25 U of PrimeSTAR GXL DNA Polymerase (TaKaRa Bio Inc.). The reaction profile was as follows: denaturation for 2 min at 98 °C, by 30 cycles of denaturation for 10 s at 98 °C, annealing for 15 s at 60 °C, and extension for 30 s or 1–3 min at 68 °C, and then a final extension at 72 °C for 5 min. Nucleotide sequences were determined on an ABI 3500xL genetic analyzer (Applied Biosystems). We determined each gene sequence using the genomic DNA from two to six males. In the event that a common substitution was identified in all males, we validated the male specificity of the substitution by verifying the absence of polymorphism at the same site in four females.

The equality of evolutionary rate between neo-X and neo-Y sequences was tested by Tajima’s relative rate test (MEGA 5.2.2) [40] with the autosomal homologs in mouse, which was more closely related species to *T. muenninki* than rat. The dN/dS ratios, which were indicator used to detect the variation in selection pressure on genes, were estimated by the branch model with CODEML program in PAML ver. 4.8 [41, 42]. This analysis was carried out based on the tree topology of the ML tree constructed by using MEGA 5.2.2. For instance, the two-ratio model of category 3 assumes that the branches of interest (both neo-X and neo-Y branch) have a dN/dS ratio that is different from the background ratio. The three-ratio model assumes that the ratios for neo-X and neo-Y branches are different and both are different from the background ratio. The above models were compared using the likelihood ratio test to examine interesting hypotheses.

## Availability of supporting data

The sequences of *T. muenninki* neo-sex–linked genes determined in this study are available on GenBank (accession numbers AB984629-AB984775). The sequence alignment of each gene among *T. muenninki*, mouse, rat, and guinea pig used in this study are included in Additional file 3.

## Additional files

Additional file 1: Table S1. List of BAC clones screened for FISH analysis in this study. **Table S2.** List of genes located on the three regions of neo-sex chromosomes in *T. muenninki*. **Table S3.** Comparison of G + C content and nucleotide substitution frequency in three regions among *T. muenninki*, mouse, and rat with guinea pig as outgroup. **Table S4.** Frequencies (%) of nucleotide substitutions in sites preceded by C or followed by G in *T. muenninki* neo-sex chromosomal segments and their autosomal homologs in mouse and rat. **Table S5.** Detection of male-specific substitutions in 24 genes located on the neo-sex chromosomes. **Table S6.** Male-specific substitutions observed in the pericentromeric region of the *T. muenninki* neo-Y chromosome. **Table S7.** Resulti of tests of the differences in d_N_/d_S_ ratios among *T. muenninki* neo-X, neo-Y, and other branches. **Table S8.** G + C content and frequency of nucleotide substitutions in *T. muenninki* neo-sex chromosomes and their homologous region in mouse. **Table S9.** List of BAC clones identified for neo-sex sequence determination. **Table S10.** Results of next-generation sequencing on an Illumina HiSeq, using a 100 bp paired-end protocol and *de novo* assembly. **Table S11.** Results of next-generation sequencing on an Illumina MiSeq, using a 300 bp paired-end protocol and *de novo* assembly. (ZIP 110 kb) Additional file 2: Figure S1. Frequency distribution of relative rates of A/T → G/C and G/C → A/T substitutions in *T. muenninki*, mouse, and rat. Each relative rate was estimated from the combined sequences of coding (3rd codon only) and noncoding sites of each gene located on the peritelomeric (a) and pericentromeric (b) regions of *T. muenninki* neo-sex chromosomes and the corresponding autosomal regions of mouse and rat. The horizontal axis shows the log of the ratio of A/T → G/C substitutions to G/C → A/T substitutions, and the vertical axis shows the number of genes. Black bar: *T. muenninki*; white bar: *M. musculus*; and gray bar: *R. norvegicus*. **Figure S2.** Comparison of nucleotide sequences of *TXNDC11* between *T. muenninki* neo-X and neo-Y. Single base insertion site in neo-Y–linked sequence was indicated by gray box. The sequences were partially skipped in TMU (2407 bp) and MMU (2410 bp), and the skipped site is shown by double slashes. The accession number for *Txndc11* gene from mouse is NM_029582. TMU: *T. muenniniki*, MMU: *M.musculus.* **Figure S3.** The estimation of nucleotide substitution directions in the three species by the maximum parsimony principle. TMU: *T. muenninki*, MMU: *M. musculus*, RNO: *R. norvegicus*. (ZIP 342 kb) Additional file 3:
**File_1 and File_2 include each gene sequence alignment of non-coding and coding region among** ***T. muenninki*** **, mouse, and rat, respectively. File_3** includes each gene sequence alignment of coding region among the three species with guinea pig. (DOC 21 kb)

## Abbreviations

PAR: Pseudoautosomal region
gBGC: GC-biased gene conversion
Zoo-FISH: Cross-species fluorescence in situ hybridization
MYA: Million years ago
FISH: Fluorescence in situ hybridization
BAC: Bacterial artificial chromosome
ORF: Open reading frame
d_N_: Non-synonymous substitution rate
d_S_: Synonymous substitution rate
XAR: Autosome fused to the X chromosome
DIG: Digoxigenin
DAPI: 4‘, 6-diamidino-2-phenylindole
NGS: Next-generation sequencing
