# Supplementary figures and images for: Initiation of recombination suppression and PAR formation during the early stages of neo-sex chromosome differentiation in the Okinawa spiny rat, Tokudaia muenninki
Source: BMC Evol Biol. 2015 Oct 29;15:234. doi: 10.1186/s12862-015-0514-y (PMC4625939; doi:10.1186/s12862-015-0514-y)

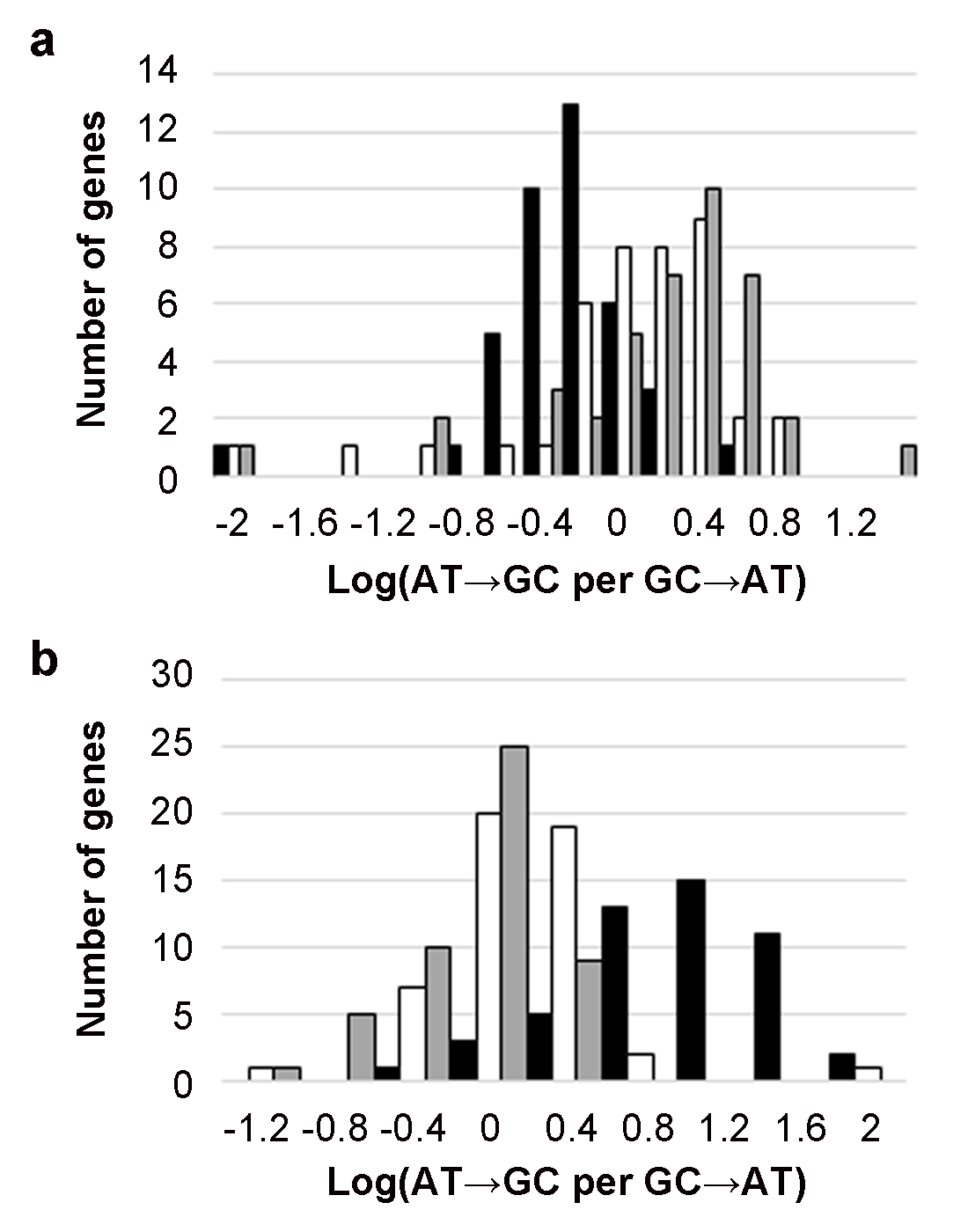

Supplement: Additional file 2: Figure S1. — Frequency distribution of relative rates of A/T → G/C and G/C → A/T substitutions in T. muenninki, mouse, and rat. Each relative rate was estimated from the combined sequences of coding (3rd codon only) and noncoding sites of each gene located on the peritelomeric (a) and pericentromeric (b) regions of T. muenninki neo-sex chromosomes and the corresponding autosomal regions of mouse and rat. The horizontal axis shows the log of the ratio of A/T → G/C substitutions to G/C → A/T substitutions, and the vertical axis shows the number of genes. Black bar: T. muenninki; white bar: M. musculus; and gray bar: R. norvegicus. Figure S2. Comparison of nucleotide sequences of TXNDC11 between T. muenninki neo-X and neo-Y. Single base insertion site in neo-Y–linked sequence was indicated by gray box. The sequences were partially skipped in TMU (2407 bp) and MMU (2410 bp), and the skipped site is shown by double slashes. The accession number for Txndc11 gene from mouse is NM_029582. TMU: T. muenniniki, MMU: M.musculus. Figure S3. The estimation of nucleotide substitution directions in the three species by the maximum parsimony principle. TMU: T. muenninki, MMU: M. musculus, RNO: R. norvegicus. (ZIP 342 kb) [file 12862_2015_514_MOESM2_ESM.zip › Murata_FigS1.tif]

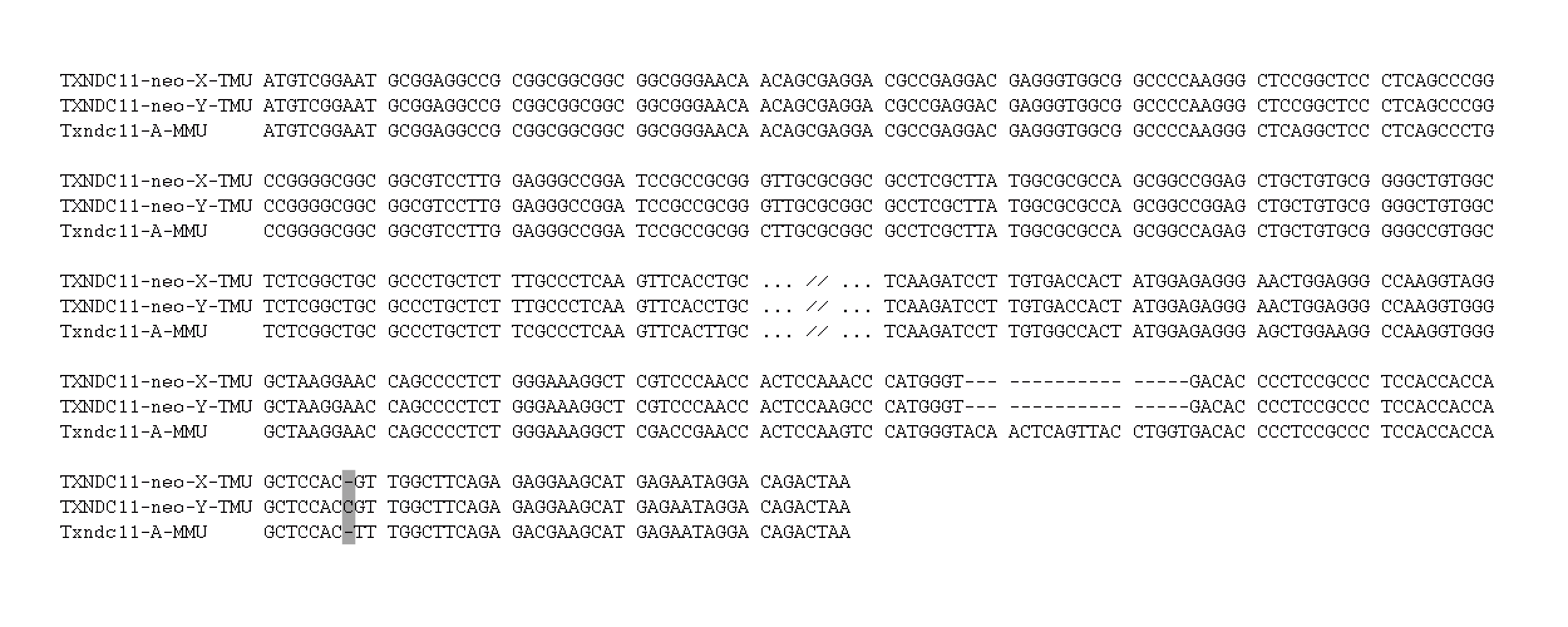

Supplement: Additional file 2: Figure S1. — Frequency distribution of relative rates of A/T → G/C and G/C → A/T substitutions in T. muenninki, mouse, and rat. Each relative rate was estimated from the combined sequences of coding (3rd codon only) and noncoding sites of each gene located on the peritelomeric (a) and pericentromeric (b) regions of T. muenninki neo-sex chromosomes and the corresponding autosomal regions of mouse and rat. The horizontal axis shows the log of the ratio of A/T → G/C substitutions to G/C → A/T substitutions, and the vertical axis shows the number of genes. Black bar: T. muenninki; white bar: M. musculus; and gray bar: R. norvegicus. Figure S2. Comparison of nucleotide sequences of TXNDC11 between T. muenninki neo-X and neo-Y. Single base insertion site in neo-Y–linked sequence was indicated by gray box. The sequences were partially skipped in TMU (2407 bp) and MMU (2410 bp), and the skipped site is shown by double slashes. The accession number for Txndc11 gene from mouse is NM_029582. TMU: T. muenniniki, MMU: M.musculus. Figure S3. The estimation of nucleotide substitution directions in the three species by the maximum parsimony principle. TMU: T. muenninki, MMU: M. musculus, RNO: R. norvegicus. (ZIP 342 kb) [file 12862_2015_514_MOESM2_ESM.zip › Murata_FigS2.tif]

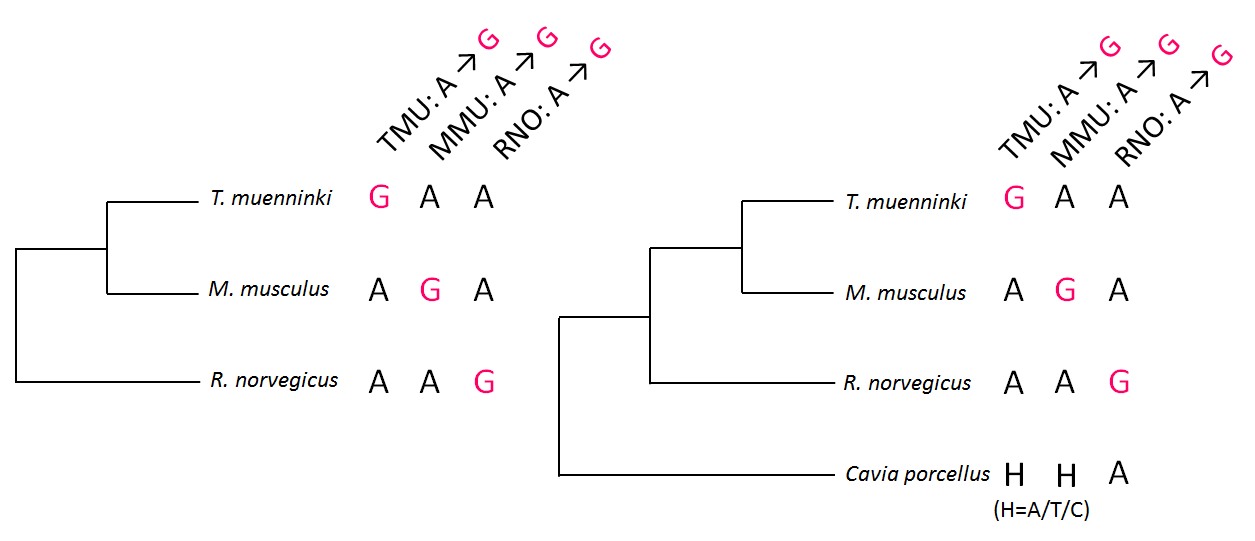

Supplement: Additional file 2: Figure S1. — Frequency distribution of relative rates of A/T → G/C and G/C → A/T substitutions in T. muenninki, mouse, and rat. Each relative rate was estimated from the combined sequences of coding (3rd codon only) and noncoding sites of each gene located on the peritelomeric (a) and pericentromeric (b) regions of T. muenninki neo-sex chromosomes and the corresponding autosomal regions of mouse and rat. The horizontal axis shows the log of the ratio of A/T → G/C substitutions to G/C → A/T substitutions, and the vertical axis shows the number of genes. Black bar: T. muenninki; white bar: M. musculus; and gray bar: R. norvegicus. Figure S2. Comparison of nucleotide sequences of TXNDC11 between T. muenninki neo-X and neo-Y. Single base insertion site in neo-Y–linked sequence was indicated by gray box. The sequences were partially skipped in TMU (2407 bp) and MMU (2410 bp), and the skipped site is shown by double slashes. The accession number for Txndc11 gene from mouse is NM_029582. TMU: T. muenniniki, MMU: M.musculus. Figure S3. The estimation of nucleotide substitution directions in the three species by the maximum parsimony principle. TMU: T. muenninki, MMU: M. musculus, RNO: R. norvegicus. (ZIP 342 kb) [file 12862_2015_514_MOESM2_ESM.zip › Murata_FigureS3.tif]
